# Inhibition of mitochondrial phosphate carrier prevents high phosphate-induced superoxide generation and vascular calcification

**DOI:** 10.1038/s12276-023-00950-0

**Published:** 2023-03-01

**Authors:** Nhung Thi Nguyen, Tuyet Thi Nguyen, Ha Thu Nguyen, Ji-Min Lee, Min-Ji Kim, Xu-Feng Qi, Seung-Kuy Cha, In-Kyu Lee, Kyu-Sang Park

**Affiliations:** 1 Department of Physiology, Yonsei University Wonju College of Medicine, Wonju, Korea; 2 Mitohormesis Research Center, Yonsei University Wonju College of Medicine, Wonju, Korea; 3 Medical Doctor Program, College of Health Sciences, VinUniversity, Hanoi, Vietnam; 4 Internal Medicine Residency Program, VinUniversity, Hanoi, Vietnam; 5 Department of Internal Medicine, School of Medicine, Kyungpook National University, Kyungpook National University Hospital, Daegu, Korea; 6 Key Laboratory of Regenerative Medicine, Ministry of Education, Department of Developmental and Regenerative Biology, Jinan University, Guangzhou, China

**Keywords:** Calcification, Mitochondria

## Abstract

Vascular calcification is a serious complication of hyperphosphatemia that causes cardiovascular morbidity and mortality. Previous studies have reported that plasmalemmal phosphate (Pi) transporters, such as PiT-1/2, mediate depolarization, Ca^2+^ influx, oxidative stress, and calcific changes in vascular smooth muscle cells (VSMCs). However, the pathogenic mechanism of mitochondrial Pi uptake in vascular calcification associated with hyperphosphatemia has not been elucidated. We demonstrated that the phosphate carrier (PiC) is the dominant mitochondrial Pi transporter responsible for high Pi-induced superoxide generation, osteogenic gene upregulation, and calcific changes in primary VSMCs isolated from rat aortas. Notably, acute incubation with high Pi markedly increased the protein abundance of PiC via ERK1/2- and mTOR-dependent translational upregulation. Genetic suppression of PiC prevented Pi-induced ERK1/2 activation, superoxide production, osteogenic differentiation, and vascular calcification of VSMCs in vitro and aortic rings ex vivo. Pharmacological inhibition of mitochondrial Pi transport using butyl malonate (BMA) or mersalyl abolished all pathologic changes involved in high Pi-induced vascular calcification. BMA or mersalyl also effectively prevented osteogenic gene upregulation and calcification of aortas from 5/6 subtotal nephrectomized mice fed a high-Pi diet. Our results suggest that mitochondrial Pi uptake via PiC is a critical molecular mechanism mediating mitochondrial superoxide generation and pathogenic calcific changes, which could be a novel therapeutic target for treating vascular calcification associated with hyperphosphatemia.

## Vascular disease: Phosphate transport protein implicated in blood vessel stiffening

Drugs that block the transport of phosphate ions into mitochondria, the ‘powerhouses’ of the cell, could prevent life-threatening stiffening of blood vessel walls. Using smooth muscle cells taken from rat aortas, Kyu-Sang Park of Yonsei University Wonju College of Medicine, South Korea, and colleagues showed how mitochondrial uptake of phosphate via phosphate transport proteins triggered toxic metabolite generation, increased activity of bone formation genes and other reactions that collectively drive the deposition of minerals, leading to vascular stiffening. Suppression of the proteins’ activity, either using drug-like compounds or by genetic means, reduced these pathological changes in mice. The findings highlight the therapeutic potential of such an approach in people with elevated serum phosphate leading to calcium build-up within their vessel walls, a major risk factor for cardiovascular disease.

## Introduction

Hyperphosphatemia is the most common complication in patients with chronic kidney disease (CKD), particularly in those with end-stage renal disease with severely reduced kidney function^1,2^. In the initial and middle stages of CKD, the serum phosphate (Pi) level actively fluctuates with Pi overload several times a day but is maintained within a normal range, likely due to a compensatory decline in tubular reabsorption controlled by enhanced production of parathyroid hormone and fibroblast growth factor 23 (FGF23)^3,4^. However, disturbances in Pi homeostasis could be responsible for the development of cardiovascular diseases in patients with CKD, including medial calcification, which is associated with mortality risk^5,6^.

High extracellular Pi stimulates the transformation of vascular smooth muscle cells (VSMCs) into a synthetic phenotype and induces secretion of matrix vesicles, leading to a calcified state^7^. Moreover, activation of the apoptotic pathway with apoptotic vesicle release by high Pi greatly contributes to the dysfunction of VSMCs, resulting in progressive vascular calcification^8–10^. We previously demonstrated that the plasmalemmal Pi transporters PiT-1 and PiT-2 are critical for Pi-induced depolarization of the plasma membrane, leading to cytosolic Ca^2+^ increase, and are partly responsible for oxidative stress and calcific changes in VSMCs^11^. High-Pi exposure causes cytosolic Pi overload, thereby accelerating mitochondrial Pi uptake and subsequently inducing mitochondrial oxidative stress in VSMCs^12^. Notably, mitochondrial reactive oxygen species (ROS) promote the nuclear translocation of NF-κB and upregulate osteogenic proteins, such as runt-related transcription factor 2 (Runx2), osteopontin (OPN), and alkaline phosphatase (ALP)^13^. Consistently, a mitochondrial antioxidant can successfully prevent osteogenic differentiation and apoptotic progression induced by high Pi^11,13^; however, to the best of our knowledge, the role of mitochondrial Pi transport in the pathogenesis of mitochondrial oxidative stress and vascular calcification has not been elucidated.

Several candidate mitochondrial Pi transporters have been proposed, including phosphate carrier (PiC)^14^, dicarboxylate carrier (DIC)^15^, Mg-ATP/Pi transporter (Mg-ATP/Pi)^16^, and uncoupling protein 2 (UCP2)^17^. PiC (Slc25a3) is primarily found in the heart and skeletal muscle^14,18^, whereas DIC is present mainly in the liver, kidneys, and white adipose tissue^15,19,20^. Mitochondrial Pi transport is essential for ATP synthesis and facilitates mitochondrial calcium uptake via the mitochondrial calcium uniporter^21^. However, under pathological conditions, mitochondrial Pi uptake can induce Pi overload, leading to mitochondrial dysfunction and further damage to cells and tissues. Given the understanding that mitochondrial Pi overload may participate in generating mitochondrial ROS and intensifying calcific progression, in the present study, we investigated whether inhibiting mitochondrial Pi transport would block the detrimental intracellular signaling and vascular calcification caused by elevated Pi.

## Materials and methods

### Ethics statement

Institutional Review Board Statement: All protocols and animal experimental procedures were performed according to the Guidelines for Animal Care and Use of Yonsei University Wonju College of Medicine (YWC-200520-3). The animals used in the study were acquired and cared for according to the guidelines of the National Institutes of Health Guide for the Care and Use of Laboratory Animals. All animals were housed in a specific pathogen-free environment under an inverse 12-h light/dark cycle and controlled stable temperature. Three to four mice were kept in one large cage with free access to food and water. The animals were simultaneously randomized into different treatment groups.

### Isolation of pVSMCs

Primary VSMCs (pVSMCs) were prepared from the thoracic aortas of 6-week-old male Sprague–Dawley (SD) rats (150–200 g) (DBL, Eumseong, Korea). Rats were anesthetized with an intraperitoneal injection of ketamine (80 mg/kg) and xylazine (40 mg/kg) under sterile conditions according to the methods in a previous report^22^. The rats were then placed in the supine position, and their chests were opened. The thoracic aorta of each rat was removed and transferred to a culture dish containing cold phosphate-buffered saline (PBS). The rats were then euthanized by cervical dislocation. Next, following the removal of the fat tissue around the thoracic aorta, the aorta was cut into 2 mm tissue blocks and transferred to a 25 cm^2^ flask with its luminal surface in contact with the flask wall. To assist adherence of the explanted tissue to the plastic culture substrate, the T-flask was kept upright for 2 h in the incubator and then carefully positioned horizontally so that the medium covered the aortic tissues. The explants were not disturbed for 4 days. Cells were trypsinized and seeded into a 60 mm cell culture dish. VSMCs from passage 3 to passage 8 were used (Supplementary Fig. 1).

### Ex vivo culture of rat thoracic aortic rings

A ring-culture assay was performed ex vivo. Briefly, 6-week-old male SD rats (150–200 g) (DBL) were anesthetized and euthanized as described above. The thoracic aorta was isolated and cut into 3 mm sections under sterile conditions. After careful removal of the surrounding fat tissue, all explants were incubated in calcification medium (Dulbecco’s modified Eagle’s medium) with a final Pi concentration of 4 mM for 7 days with or without drugs or siRNA treatment.

### Mouse CKD model

Subtotal nephrectomy was performed on 8-week-old C57BL/6 mice (DBL) in two steps according to a validated protocol^23^. Subtotal nephrectomy was performed using isoflurane anesthesia (1.5–2%). In the first step, the left kidney was ligated using a 3-0 silk suture string approximately 0.4 cm from the superior toward the middle of the pylorus and 0.4 cm from the inferior portion toward the pylorus. Mice were administered antibiotics and painkillers every 12 h for the first 3 days after surgery. Seven days later, the right kidney was completely removed. Sham-operated mice were used as the controls. Three days after the second surgery, the rodent diet was switched from a normal chow diet to a high-Pi (2.1%) diet, which was sustained for 12 weeks. Simultaneously, all animals were randomized to different treatment groups and administered an intraperitoneal injection of inhibitors every 3 days. After all experiments were completed, the mice were euthanized by cervical dislocation, and tissues were collected for analysis.

### Alizarin Red S and von Kossa staining

Following incubation in calcification medium, VSMCs were fixed with 4% paraformaldehyde (PFA; Sigma, St. Louis, MO, USA) for 10 min and washed twice with PBS. Alizarin Red S (2%; ScienCell, Carlsbad, CA, USA) was added to the cells, which were incubated for 30 min and then rinsed with deionized distilled water to visualize Ca^2+^ precipitation. The calcium deposits were dissolved using dimethylsulfoxide (Sigma), and the absorbance of the colored solution was measured at 450 nm using a microplate spectrophotometer (Epoch, Bio-Tek, Winooski, VT, USA).

For staining, tissues were embedded in paraffin and cut into 5-μm-thick sections. Thereafter, the sections were deparaffinized with two washes of xylene for 10 min each. The tissues were rehydrated with two washes of absolute alcohol for 5 min, 95% alcohol for 3 min, and 70% alcohol for 3 min and then rinsed with running tap water for 10 min. For von Kossa staining, tissues were incubated with silver nitrate solution for 60 min under UV light. Thereafter, the tissues were washed with distilled water and placed in sodium thiosulfate solution for 5 min with shaking. For calcification staining, tissue was stained with Alizarin Red S (ScienCell, Carlsbad, USA) for 5 min. Thereafter, the washing steps were repeated, and the sections were dehydrated using 95% alcohol and two washes of absolute alcohol for 5 min. The sections were further washed twice with xylene for 5 min each. Finally, the stained tissues were preserved with a xylene-based mounting medium.

### Micro-computed tomography (micro-CT) imaging

High-resolution micro-CT was performed using a Quantum FX system (Perkin Elmer, Waltham, MA, USA). Vascular tissues and femur heads were imaged with an isotropic voxel resolution of 19 µm. The voltage used was 90 kV, and the current was 180 µA. The total scan time was 3 min per sample. The scan files were automatically reconstructed using Quantum FX (Perkin Elmer).

### Micro-CT image processing

The micro-CT data of vascular tissues and femur heads were analyzed using Analyze 12.0 software. Noise in the reconstructed images was reduced by applying a median filter, and postprocessing was performed using an adaptive histogram equalization filter to emphasize the contrast between calcified and normal tissue. After thresholding, detecting differences in tissue density enabled the identification of areas of calcification within soft tissues. Quantification was performed by calculating the average threshold signal intensity using the software.

### Quantification of calcium content in aortic rings

To quantify the calcium content in rat aortic rings, after the indicated treatment, tissues were incubated overnight at 37 °C in 0.6 M HCl. Thereafter, the supernatant was collected in a 1.5 mL tube, and the calcium content was determined using a Quantichrom Calcium assay Kit (BioAssay Systems, cat: DICA-500) following the manufacturer’s protocol.

### Quantitative PCR

Total RNA was isolated from pVSMCs and aortas using a Hybrid RTM RNA purification kit (GeneAll, Seoul, Korea, #305-101). The purity of RNA was checked by measuring the absorbance ratio at 260 nm and 280 nm. Complementary DNA was synthesized from total RNA using ReverTraAce® qPCR RT Master Mix with gDNA Remover (Toyobo, Osaka, Japan, # FSQ301). Quantitative RT‒PCR was performed using an Applied Biosystems QuantStudio 6 Flex Real-Time PCR system and SYBR Green I (AB Bioscience, Concord, MA, USA, #AB4367659). The sequences of the primers are listed in Supplementary Table 1. All quantitative PCR data consist of the average values of triplicates and were evaluated using the delta-delta-CT method with *Actb* as a reference gene. The mRNA expression levels were normalized to the control level to show relative changes.

### Immunoblotting

After the indicated treatment, cells were washed three times with PBS and lysed with cold RIPA buffer (Pierce, Waltham, MA, USA, #89900) containing a protease inhibitor (Roche, Basel, Switzerland, #4693159001) and phosphatase inhibitor cocktail (Roche, #4906837001). Cell lysates were collected in 1.5 mL tubes and centrifuged at 13,000 rpm for 30 min. The supernatants were collected without disturbing the pellets, and the protein levels were determined using a bicinchoninic acid assay kit (Pierce, #23223). The supernatants were subjected to sodium dodecyl sulfate–polyacrylamide gel electrophoresis, and the resolved protein bands were transferred onto polyvinylidene difluoride membranes (Merck Millipore, Billerica, MA, USA, #IPH00010). The membranes were incubated with 5% bovine serum albumin or 6% skim milk for 1 h at room temperature to eliminate nonspecific binding of antibodies. After blocking, the membranes were washed three times with 0.1% TBST for 10 min each and incubated with primary antibodies against the following proteins overnight at 4 °C: β-Actin (Abcam, Cambridge, MA, USA, #ab6276), p-ERK1/2 (Cell Signaling, Danvers, MA, USA, #4370P), ERK1/2 (Cell Signaling, #9102), p-p70S6K (Cell Signaling, #9205S), and p70S6K (Cell Signaling, #9202). The primary antibody for PiC was generously provided by Prof. A. P. Halestrap (University of Bristol, Bristol, UK). Following primary antibody incubation, the membranes were washed three times and incubated with secondary anti-rabbit (Invitrogen, Carlsbad, CA, USA, #31460) or anti-mouse horseradish peroxidase-conjugated antibodies (Invitrogen, #31450) for 1 h at room temperature. The blots were developed using ECL detection reagent (Amersham BioSciences, Little Chalfont, UK, #RPN2235/2232). Each blot was quantified using ImageJ software. The protein level was normalized to that of β-actin and expressed as the relative change compared to the control.

### Isolation of cytosolic and mitochondrial fractions

Primary VSMCs were washed twice with PBS and harvested using a scraper. The cell pellet was resuspended in ice-cold homogenization buffer (75 mM sucrose, 225 mM mannitol, 0.1 mM EGTA, 30 mM Tris-HCl; pH 7.4), homogenized with a 26G syringe, and centrifuged at 500 × *g* for 5 min. The pellet was discarded, and the supernatant containing crude mitochondria and cytosol was centrifuged at 10,000 × *g* for 20 min. The supernatant was collected as the cytosolic fraction. The pellet was washed with ice-cold homogenization buffer and centrifuged again to collect the crude mitochondrial fraction.

### Mitochondrial ROS measurement

To evaluate mitochondrial ROS, cells were incubated with mitoSOX (5 μM; Invitrogen, #M36008) in Krebs–Ringer bicarbonate buffer for 20 min at 37 °C. Following incubation, the cells were washed twice with the same buffer. Fluorescence images (excitation/emission: 510/580 nm) were captured and analyzed using MetaMorph software (Molecular Devices, San Jose, CA, USA).

### siRNA transfection

To silence the expression of PiC, DIC, UCP2, slc25a23, slc25a24, and slc25a25, siGENOME Smartpool siRNA duplexes were purchased from Dharmacon-Horizon Discovery (Cambridge, UK). SiRNA was transfected using the DharmaFECT-1 siRNA transfection reagent (#T-2001-03; Thermo Fisher Scientific, Waltham, MA, USA). Cells and tissues were transfected with siRNA in DharmaFECT-1 in Opti-MEM (Gibco, #31985-070) at 8 h after VSMC seeding or 2–3 h after isolation of rat aortic rings. The knockdown efficiency was confirmed by western blotting at 72 h or qPCR at 48 h after siRNA transfection.

### Mitochondrial membrane potential measurement

To determine the mitochondrial membrane potential (ΔΨm), VSMCs were loaded with JC-1 (300 nM; Invitrogen, #T3168) for 30 min at 37 °C. *Staphylococcus aureus* α-toxin (Sigma, #H9395) was used to permeabilize the cell membranes, and the cells were placed in intracellular buffer solution (140 mM KCl, 5 mM NaCl, 7 mM MgSO_4_, 1 mM KH_2_PO_4_, 1.65 mM CaCl_2_, 10.2 mM EGTA, 20 mM HEPES, pH 7.0) for 10 min in an incubator. Mitochondrial membrane potential was estimated based on the ratio of J-aggregate (540 nm excitation and 590 nm emission) to JC-1 monomer (490 nm excitation and 540 nm emission) fluorescence intensities.

### Immunofluorescence staining

VSMCs on coverslips were washed twice with PBS and fixed with 4% PFA or cold absolute methanol for 10 min at room temperature. Triton X (0.1%) in PBS was administered to permeabilize the cells for 10 min. Thereafter, the cells were carefully washed three times with PBS and blocked with 5% normal goat serum in PBS for 1 h at room temperature. The cells were incubated overnight in a cold chamber with PiC antibody (1:1000 dilution). The next day, the cells were incubated with the secondary antibody Alexa Fluor 488 goat anti-rabbit IgG (1:100 dilution, Invitrogen, #A11008). The nuclei were stained with 1 μg/mL 4’,6’-diamidino-2-phenylindole (DAPI; Invitrogen, #D1306) for 5 min and mounted on a glass slide.

Following the removal of fat, murine thoracic aortic tissues were fixed in 4% PFA, and paraffin sections were prepared. Thereafter, the tissues were dipped in Neoclear and a series of ethanol solutions (95–90–80–70%). Next, the slides were washed with deionized distilled water (3DW) and PBS and underwent antigen retrieval using citrate buffer (10 mM, pH 6) at 95 °C for 20 min. Thereafter, the tissues were blocked with 0.3% H_2_O_2_ for 30 min, washed with 3DW and PBST, and blocked again with normal goat serum. The tissues were incubated with the primary antibody anti-Runx2 (Abcam, Cat # ab76956) in 2% normal goat serum overnight at 4 °C and washed with PBST. Thereafter, the slides were incubated with the secondary antibody and washed four times with PBST. The nuclei were stained with DAPI for 5 min, and the tissues were mounted. Fluorescence pictures were obtained using a confocal laser-scanning microscope (LSM 800; Zeiss, Oberkochen, Germany). The negative control was prepared following all the above steps without primary antibody incubation.

### Measurement of blood urea nitrogen/creatinine/Pi/FGF23

The serum concentrations of blood urea nitrogen, creatinine, and Pi were measured using a photometric method (FUJI FDC 3500i, Sysmex, Kobe, Japan). Serum FGF23 was determined using a mouse FGF-23 DuoSet ELISA kit (Cat DY2629-05 and DY008; R & D Systems, Minneapolis, MN, USA) according to the manufacturer’s instructions.

### Statistical analyses

The results are expressed as the mean ± SEM, with n values stated in the figure legends. Technical replicates were used to ensure the reliability of each value. The differences among groups were assessed using one-way analysis of variance (ANOVA), followed by Tukey’s multiple comparison test. *P* values < 0.05 were considered to indicate significance. All statistical analyses were performed using GraphPad Prism (Version 9.1.0.221, GP9-1988787-RLRM-FA1F1, GraphPad Software, San Diego, CA).

## Results

### High extracellular Pi increases mitochondrial phosphate carrier abundance and vascular calcification in VSMCs

**Fig. 1:**
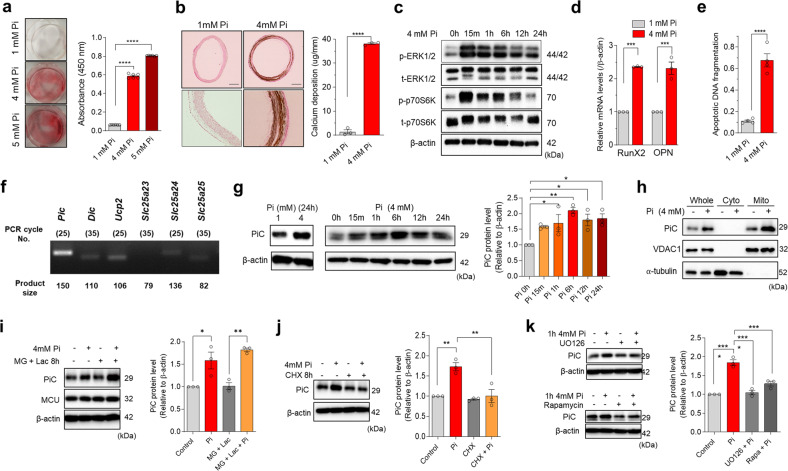
High extracellular phosphate (Pi) increases the abundance of Pi carrier (PiC) and elicits vascular calcification in primary vascular smooth muscle cells (pVSMCs) and rat aortic rings. **a** Calcification of pVSMCs was visualized by Alizarin Red S staining upon Pi treatment for 48 h (# experiments; *n* = 6). **b** Thoracic aortic rings isolated from SD rats were cultured in calcifying medium (1 mM and 4 mM Pi) for 7 days, and calcium deposition was detected using von Kossa staining with a dark brown color (# rats; *n* = 4). **c** Time-dependent activation of ERK1/2 and mTOR by 4 mM Pi incubation in pVSMCs was determined using western blotting (# experiments; *n* = 3). **d** The transcript levels of osteogenic genes were estimated using quantitative PCR upon 1 mM and 4 mM Pi treatment for 48 h (# experiments; *n* = 3). **e** Apoptotic DNA fragmentation of pVSMCs was measured after 48 h of incubation with medium containing high levels of Pi (# experiments; *n* = 4). **f** Endpoint PCR analysis to check the expression of the mitochondrial phosphate transporters PiC, DiC, UCP2, slc25a23, alc25a24, and slc25a25 in pVSMCs (# experiments; *n* = 3). **g** Time-dependent increase in PiC protein expression after high-Pi incubation as revealed by immunoblotting (# experiments; *n* = 3). **h** PiC expression and upregulation by high Pi in cytosolic and mitochondrial fractions of pVSMCs. **i** Effects of proteasomal inhibitors (MG132 + lactacystin) on the upregulation of PiC after 1 h of treatment with 4 mM Pi (# experiments; *n* = 3). **j** Protein expression of PiC in pVSMCs after preincubation with CHX for 8 h followed by 1 h of high Pi culture (# experiments; *n* = 3). **k** Effects of UO126 and rapamycin on the increase in PiC after treatment of pVSMCs with elevated Pi-containing medium (# experiments; *n* = 3). The data were analyzed by either two-tailed Student’s t test or one-way ANOVA. *P < 0.05; **P < 0.01; ***P < 0.001; ****P < 0.0001.

To investigate the importance of mitochondrial Pi transport in high Pi-induced vascular calcification, we constructed calcification models using pVSMCs (in vitro) and cultured rat aortic rings (ex vivo). Maintenance of pVSMCs in high Pi-containing medium for 2 days markedly increased calcification, which was visualized using Alizarin Red S staining (Fig. 1a). Calcification in the medial layer of the aortic ring was detected on day seven after high-Pi exposure (Fig. 1b). Consistent with a previous study, elevated Pi increased the phosphorylation of ERK1/2 and p70S6K downstream of mTOR (Fig. 1c)^11^. High Pi upregulated the osteogenic genes *Runx2* and *osteopontin* in pVSMCs (Fig. 1d). In addition, DNA fragmentation was augmented by high-Pi incubation, indicating activation of the apoptotic process (Fig. 1e).

Next, we examined the expression profiles of mitochondrial Pi transporters in pVSMCs. Notably, PiC was the most highly expressed Pi transporter, whereas UCP2, DIC, slc25a24, and slc25a25 showed lower expression (Fig. 1f, Supplementary Fig. 2a). Intriguingly, the protein abundance of PiC was increased under high-Pi incubation (Fig. 1g) and was detected early (15 min), continuing until 24 h. We further confirmed that PiC was exclusively expressed and upregulated in the mitochondrial fraction of pVSMCs (Fig. 1h), which could accelerate mitochondrial Pi uptake under high-Pi conditions. The transcription of PiC was not altered during 24 h of high-Pi exposure (Supplementary Fig. 2b), which indicated the possible involvement of a degradational and/or translational pathway. First, the rate of degradation and translation of PiC was independently determined. A pulse-chase assay was performed to assess the turnover rate of PiC. Cells were treated with cycloheximide (CHX), which represses the translational elongation step in eukaryotes^24^. CHX treatment quickly reduced the abundance of PiC, indicating that the translation of PiC protein was highly dynamic (Supplementary Fig. 2c). To further identify the underlying mechanism for PiC upregulation, we applied MG132 and lactacystin (LAC), both of which suppress the degradation of ubiquitin-conjugated proteins by blocking the proteolytic activity of the 26S proteasome complex^25^. The data showed negligible accumulation of PiC after treatment with MG132 and LAC in a time-dependent manner (Supplementary Fig. 2d). Thereafter, pVSMCs were treated with high Pi along with the above inhibitors. As shown in Fig. 1i, cells pretreated with MG132 and LAC continued to show an increase in PiC (1 h); however, CHX treatment completely prevented Pi-induced PiC upregulation (Fig. 1j). These results suggested that the increased abundance of PiC induced by elevated Pi was primarily triggered by translational regulation. Protein translation can be activated by mTOR and its downstream p70S6K. As shown in Fig. 1c, high-Pi incubation increased the phosphorylation of ERK1/2 and p70S6K. Therefore, we applied UO126, a MEK inhibitor, or rapamycin, an mTOR inhibitor, to examine the effect of upstream signaling on the translational regulation of PiC induced by high Pi. Inhibition of either ERK1/2 or mTOR signaling repressed the upregulation of PiC upon high-Pi treatment (Fig. 1k).

### Gene silencing of mitochondrial phosphate transport prevents high Pi-induced ROS generation and vascular calcification in pVSMCs and cultured aortic rings

**Fig. 2:**
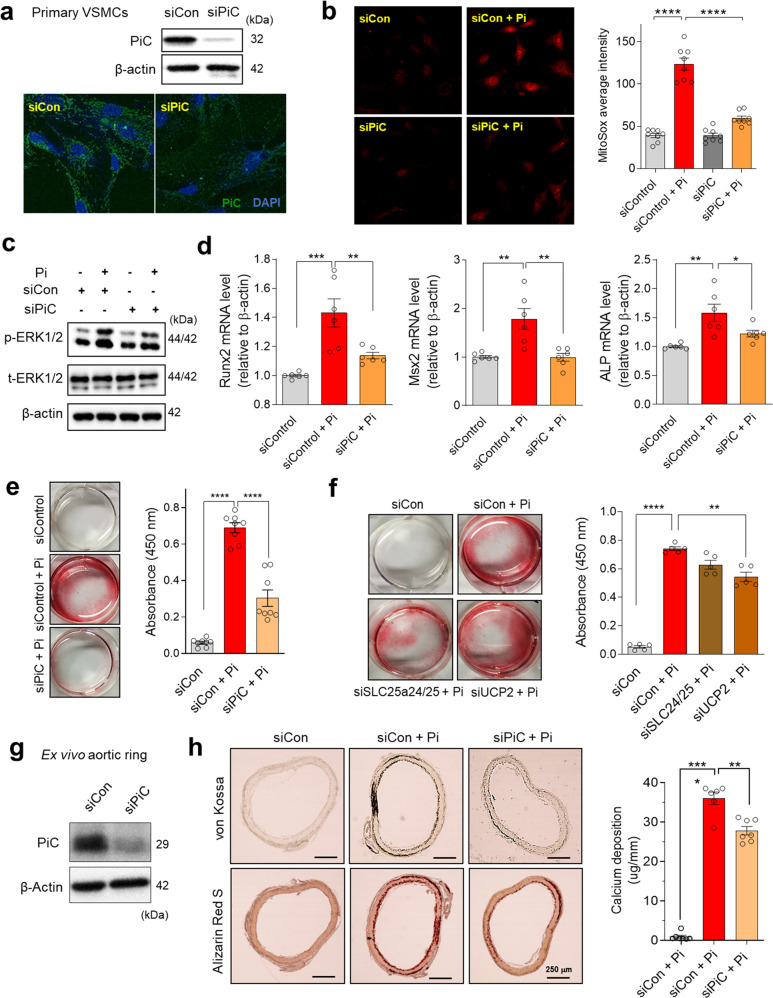
Knockdown of phosphate carrier (PiC) suppresses reactive oxygen species (ROS) generation, osteogenic differentiation, and vascular calcification caused by high Pi. **a** The knockdown efficiency of PiC in primary vascular smooth muscle cells (pVSMCs) was confirmed using immunoblotting and immunofluorescence staining. **b** Mitochondrial ROS were measured using MitoSOX to compare the mitochondrial ROS levels between control and PiC-knockdown pVSMCs. Cells on coverslips were incubated in a high Pi-containing medium for 4 h before measurement (# experiments; *n* = 9). **c** ERK1/2 activation in control and PiC-knockdown pVSMCs was detected using immunoblotting. **d** Effect of PiC gene silencing on the mRNA levels of osteogenic genes, including *RunX2*, *Msx2*, and *ALP* (# experiments; *n* = 6). **e** Calcification levels of control and PiC-knockdown pVSMCs after 48 h of high-Pi treatment. Calcium deposition was detected by Alizarin Red S staining (# experiments; *n* = 8). **f** Effects of knocking down other mitochondrial Pi transporters, including UCP2 and SLC25a24/a25, on the calcification triggered by high Pi in pVSMCs (# experiments; *n* = 5). **g** PiC knockdown efficiency in rat aortic rings cultured in Dulbecco’s modified Eagle’s medium. **h** Calcification of rat aortic rings after incubation ex vivo with a high Pi-containing medium for 7 days as visualized by Alizarin Red S or von Kossa staining (# rats; *n* = 5). The data represent the mean ± SEM and were analyzed using one-way ANOVA. *P < 0.05; **P < 0.01; ***P < 0.001; ****P < 0.0001.

As PiC showed the highest expression level among mitochondrial Pi transporters in pVSMCs (Supplementary Fig. 2a) and its protein level significantly increased with high-Pi treatment (Fig. 1g), we investigated the pathogenic role of PiC in vascular calcification using RNA interference-mediated silencing of PiC. Knockdown of PiC resulted in 80% reductions in the mRNA and protein levels (Fig. 2a and Supplementary Fig. 3a). Suppression of PiC expression inhibited mitochondrial superoxide generation (Fig. 2b) and ERK1/2 activation (Fig. 2c) after incubation with high Pi. Additionally, upregulation of osteogenic genes, such as *Runx2*, *Msx2*, and *ALP*, was blunted by knockdown of PiC (Fig. 2d). Consequently, the calcific changes in pVSMCs triggered by elevated Pi were diminished by PiC silencing (Fig. 2e). These results are consistent with those of our previous report showing that mitoTEMPO, as a mitochondrial superoxide scavenger, inhibits ERK1/2-mTOR activation, osteogenic gene upregulation, and vascular calcification^11^.

We silenced UCP2, DIC, slc25a24, and slc25a25 in pVSMCs to estimate the participation of other mitochondrial Pi transporters in calcification (Supplementary Fig. 3b–e). Among other mitochondrial Pi transporters, knockdown of UCP2 partially reduced calcification in pVSMCs to a lower level than PiC, whereas silencing of other Pi transporters did not cause any significant changes (Fig. 2f and Supplementary Fig. 3 f). The preventive effect of PiC knockdown on Pi-triggered vascular calcification was validated in cultured rat aortic rings ex vivo (Fig. 2g, h).

### Pharmacological blockade of mitochondrial phosphate transport reduces mitochondrial oxidative stress and calcification in pVSMCs

**Fig. 3:**
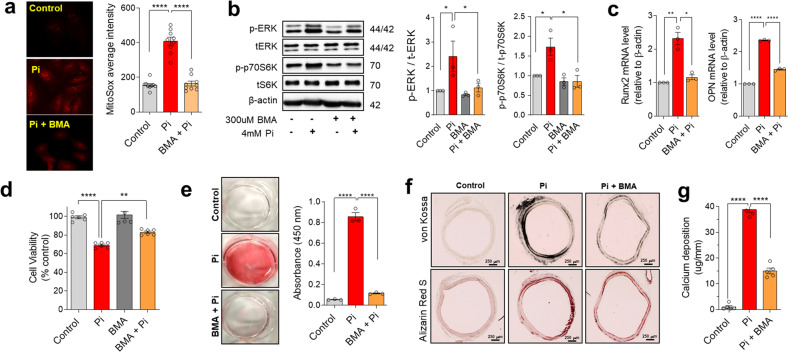
Pharmacological blockade of mitochondrial phosphate (Pi) transport prevents vascular calcification caused by high Pi. **a** Mitochondrial superoxide production in control and butyl malonate (BMA)-treated primary vascular smooth muscle cells (pVSMCs) was determined using mitoSOX (# experiments; *n* = 9). **b** Immunoblotting of ERK1/2 and p70S6K was performed (# experiments; *n* = 3). **c** The transcript levels of osteogenic genes were estimated using quantitative PCR (# experiments; *n* = 3). **d** Cell viability was evaluated using an MTT assay (# experiments; *n* = 5). **e** Calcification was quantitated using Alizarin Red S staining (# experiments; *n* = 3). **f** Calcification of rat aortic rings after high-Pi incubation ex vivo with and without BMA treatment for 7 days as visualized by Alizarin Red S or von Kossa staining. **g** The calcium content in rat aortic rings was measured using a Quantichrom Calcium Assay Kit (# rats; *n* = 5). The data were analyzed using one-way ANOVA. *P < 0.05; **P < 0.01; ****P < 0.0001.

Our data confirmed that suppression of mitochondrial Pi transporters inhibits Pi-induced vascular calcification. Although silencing of PiC showed the greatest protective activity against Pi-induced calcification, other routes for mitochondrial Pi uptake also contribute to calcific progression in pVSMCs. Instead of silencing multiple transporters, we utilized nonselective pharmacological blockers of mitochondrial Pi transporters, butylmalonate (BMA), and mersalyl. We previously demonstrated that mitochondrial Pi uptake can be effectively inhibited by BMA and mersalyl, which do not have nonspecific actions on plasmalemmal Pi transport^26^. Pretreatment with BMA prevented high Pi-induced mitochondrial membrane hyperpolarization (Supplementary Fig. 4) and mitochondrial ROS generation (Fig. 3a). Moreover, BMA considerably obstructed the ERK1/2-mTOR activation induced by high Pi (Fig. 3b). Furthermore, osteogenic gene upregulation (Fig. 3c), cytotoxicity (Fig. 3d), and calcific changes (Fig. 3e) were repressed by BMA pretreatment in pVSMCs. In the ex vivo model of cultured isolated rat aortic rings, preincubation with BMA protected against high Pi-induced calcification, as estimated using von Kossa staining, Alizarin staining (Fig. 3f), and calcium content measurement (Fig. 3g and Supplementary Fig. 5).

### Inhibiting mitochondrial Pi uptake prevents vascular calcification in a murine model of chronic renal failure

**Fig. 4:**
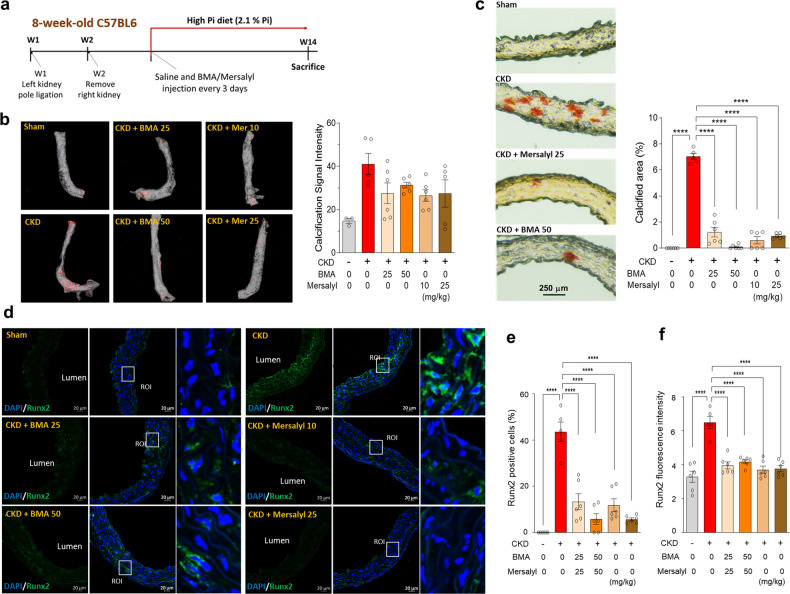
Inhibiting mitochondrial phosphate (Pi) transport attenuates vascular calcification in mice with chronic kidney disease. **a** Timeline of surgery and drug treatments in 5/6 nephrectomy mice. **b** Calcified areas in the aortic rings of 5/6 nephrectomy mice were visualized by micro-CT imaging (# mice; *n* = 3–6). **c** Alizarin Red S staining was used to identify the microcalcified areas (# mice; *n* = 5–6). **d** Immunostaining of Runx2 in microsections of mouse aortic rings, Runx2 = green, nuclei = blue. **e**, **f** Bar graph showing the number of RunX2-positive cells and the Runx2 fluorescence intensity after normalization (# mice; *n* = 5–6). The data were analyzed using one-way ANOVA. ****P < 0.0001.

To demonstrate the protective effect against medial calcification achieved in vivo by blocking mitochondrial Pi uptake, we used a CKD mouse model fed a high-Pi (2.1%) diet for 12 weeks (Fig. 4a). Male C57BL/6 mice with subtotal (5/6) nephrectomy had increased blood urea nitrogen levels with significant reductions in body weight (Supplementary Fig. 6a and 6b). Serum Pi levels were not significantly different among groups (Supplementary Fig. 6c), but the plasma FGF23 concentration was markedly increased after subtotal nephrectomy with a high-Pi diet, which was due to a compensatory response to the elevated Pi load (Supplementary Fig. 6d). Calcification of the whole aorta in this model was visualized using micro-CT imaging, which showed that treatment with BMA (25 and 50 mg/kg, IP injection every 3 days) or mersalyl (10 and 25 mg/kg) reduced the calcification signal intensity from the thoracic aorta in the CKD model (Fig. 4b). To visualize the locations of calcified deposits, Alizarin Red S staining was performed in aortic paraffin sections, which indicated that calcification developed in the medial layer of the blood vessel and calcified areas were markedly decreased in the BMA- or mersalyl-treated groups (Fig. 4c). To examine whether inhibitors of mitochondrial Pi uptake affect osteogenic protein expression in CKD mice, immunostaining of aortic tissues was performed with an anti-Runx2 antibody. As expected, Runx2 expression was greatly enhanced in the aortas of CKD mice and primarily located in the cytoplasm of VSMCs (Fig. 4d). BMA or mersalyl treatment in CKD mice decreased the number of Runx2-positive cells (Fig. 4e) and the total fluorescence intensity (Fig. 4f).

## Discussion

The present study demonstrated that a high-Pi environment increases Pi uptake into the mitochondrial matrix mainly via PiC, leading to mitochondrial superoxide generation and cell death in VSMCs. Reducing mitochondrial Pi uptake through genetic suppression of PiC inhibits mitochondrial oxidative stress, ERK1/2 activation, osteogenic gene upregulation, and calcification in vitro and ex vivo. Pharmacological blockade of PiC, which is selective for mitochondrial Pi uptake, prevents all the pathologic changes participating in high Pi-induced calcification of VSMCs, isolated aortic rings, and vascular walls of the thoracic aorta in a mouse model of CKD fed a high-Pi diet. Furthermore, we report for the first time that high extracellular Pi increases the abundance of PiC through translational activation, which can act through positive feedback to amplify mitochondrial Pi uptake under Pi-overloaded conditions. To block this vicious cycle, targeting the mitochondrial Pi transporter could be an efficient therapeutic strategy for vascular calcification associated with hyperphosphatemia.

Previous studies have suggested that high extracellular Pi exposure induces mitochondrial membrane hyperpolarization and superoxide production in different types of cells, including VSMCs^13,26,27^. In the present study, we observed the predominant role of PiC in mitochondrial Pi transport responsible for superoxide generation, which might be related to an augmented mitochondrial electrical gradient^26^. Mitochondrial Pi uptake via PiC is coupled with H^+^; thus, Pi transport driven by the proton gradient attenuates the mitochondrial chemical (pH) gradient. Hyperpolarization in response to mitochondrial Pi uptake could reflect a compensatory increase in the electrical gradient under the maintained proton motive force^27^. In addition, Pi accelerates oxidative phosphorylation by activating metabolic enzymes and respiratory chain activity, further increasing the mitochondrial electrical gradient^27^. Notably, PiC has been reported to be a critical component of the mitochondrial permeability transition pore, which can be opened by a Ca^2+^-triggered conformational change in PiC^28,29^. Furthermore, mitochondrial Pi overload primarily accelerates the opening of the mitochondrial permeability transition pore along with Ca^2+^ elevation^30^. Taken together, the findings indicate that mitochondrial Pi uptake via PiC could actively participate in high Pi-triggered apoptosis of VSMCs related to oxidative stress and Pi overload in the matrix, aggravating the progression of vascular calcification.

The pathogenic role of ERK1/2 and mTOR signaling under high-Pi conditions has been demonstrated by using inhibitors such as UO126 and rapamycin, which abolish oxidative stress, cell death, osteogenic differentiation, and calcification of VSMCs^11,12^. In particular, the plasmalemmal Pi transporters PiT-1/2 are involved in high Pi-activated ERK1/2, mTOR, and p70S6K, which in turn upregulate PiT-1/2 abundance. Additionally, high Pi stimulates plasma membrane trafficking of PiT-1/2, further accelerating Pi uptake into the cytosol^11^. The present study demonstrated that high Pi increases PiC abundance through the activation of protein translation dependent on ERK1/2-mTOR signaling. The upregulated plasmalemmal and mitochondrial Pi transporters accumulate cytosolic and mitochondrial ROS, which contribute to the release of NF-κB from IκBα, allowing it to translocate to the nuclei in VSMCs. NF-κB then acts as a transcriptional activator for numerous osteogenic genes, including *Runx2* and *OPN*, and plays a central role in vascular calcification^11,13,31^.

We observed that the concentration of extracellular Pi required to induce marked calcific changes in primary VSMCs was lower than that for the aortic smooth muscle cell line A7r5^11^. This may have originated from the difference in sensitivity to high Pi between primary and clonal cells. Notably, the plasma Pi level in patients with CKD eliciting pathologic vascular calcification is significantly lower than experimental doses of Pi in vitro since the average Pi concentration in humans is much lower than in rodents. We demonstrated that exposure to 2.6 mM Pi for 2 weeks was sufficient to trigger calcific changes in pVSMCs (Supplementary Fig. 8), suggesting that long-term Pi exposure in patients with CKD shares a pathogenic mechanism for vascular calcification with in vitro calcific changes in VSMCs.

There are several known mitochondrial Pi transporters, including PiC, DIC, UCP2, and Mg-ATP/Pi, all of which belong to the SLC25 family. These transporters are located in the inner membrane and mediate mitochondrial Pi uptake^32,33^. Through RNAi-mediated knockdown experiments for all known mitochondrial Pi transporters, we found that silencing of PiC or UCP2 showed significant protection against Pi-induced calcification. However, mitochondrial Pi uptake via a single transporter seems not to be exclusive, which could be a limitation of therapeutic strategies using genetic suppression. Instead, BMA or mersalyl, which inhibit mitochondrial Pi uptake via SLC25 transporters, elicited more efficient protection against Pi-related oxidative stress and calcification. We applied pharmacologic inhibition to an in vivo CKD mouse model fed a high-Pi diet, which showed reduced aortic calcification, as detected by micro-CT and tissue staining. Osteogenic Runx2 expression was highly increased in the aortic walls of CKD mice but returned to normal levels upon inhibition of mitochondrial Pi uptake. Notably, we confirmed that long-term treatment (16 weeks) with BMA or mersalyl did not affect the bone mineral density and structure of the mouse femoral head (Supplementary Fig. 7).

Hyperphosphatemia could be a major risk factor for the development of vascular calcification in patients with CKD or abnormal hormonal regulation. In a patient with predialysis CKD, an increase in serum Pi levels usually occurs at later stages, which often delays treatment for hyperphosphatemia^34,35^. However, in the early stage of CKD without serious elevation of serum Pi, ectopic calcification in soft tissue begins^36^, and oxidative stress is a causative factor for these pathologic changes^37^. We demonstrated that mitochondrial Pi accumulation causes mitochondrial hyperpolarization and superoxide generation, ERK1/2-mTOR activation, nuclear translocation of NF-κB, and osteogenic gene upregulation, leading to the development of vascular calcification. Moreover, PiC upregulation, as a response to external Pi load, aggravates mitochondrial Pi accumulation. Suppressing mitochondrial Pi uptake, as well as scavenging mitochondrial ROS^11^, effectively prevents osteogenic transdifferentiation and calcification. The current observations warrant further studies on mitochondrial Pi transport inhibition as a novel therapeutic strategy for vascular calcification related to chronic renal diseases.

## Supplementary information

Supplemental Information
