# Supplementary material for: Inhibition of mitochondrial phosphate carrier prevents high phosphate-induced superoxide generation and vascular calcification
Source: Exp Mol Med. 2023 Mar 1;55(3):532–40. doi: 10.1038/s12276-023-00950-0 (PMC10073177; doi:10.1038/s12276-023-00950-0)

Supplemental Information

Contents

- Supplementary Figure 1
- Supplementary Figure 2
- Supplementary Figure 3
- Supplementary Figure 4
- Supplementary Figure 5
- Supplementary Figure 6
- Supplementary Figure 7
- Supplementary Figure 8

- Supplementary Table 1

- Western blots with membranes and markers

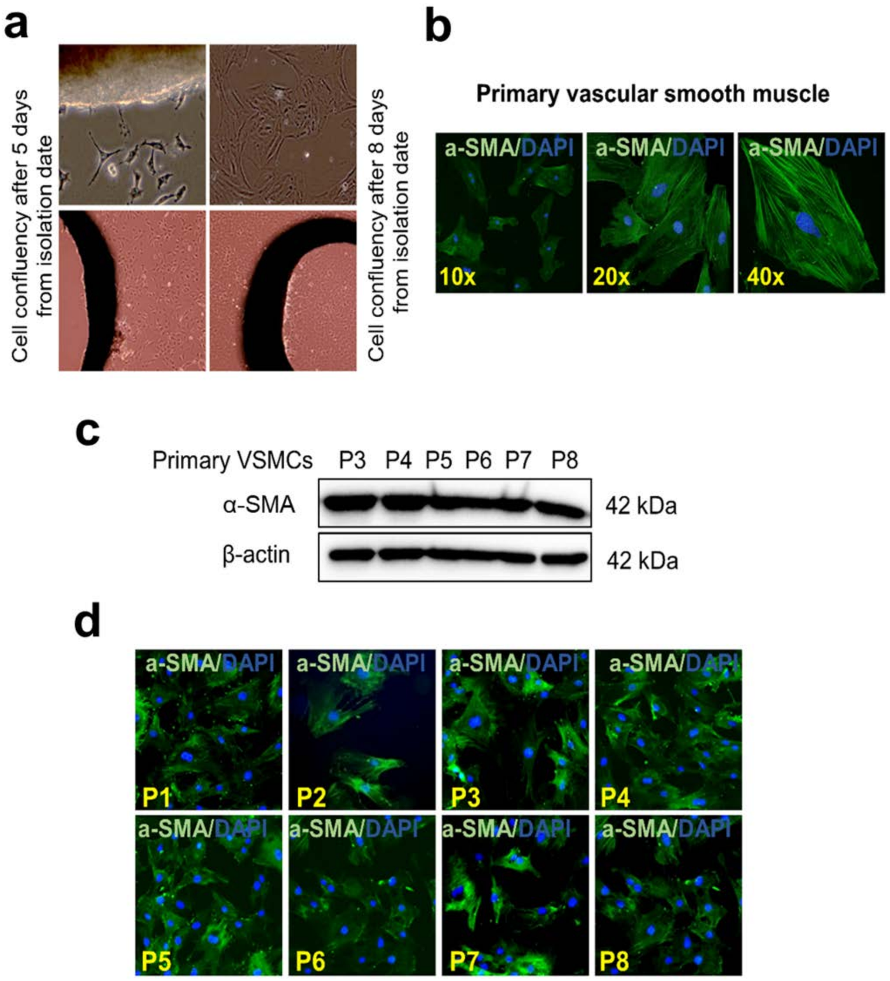

Supplementary Fig. 1. Characteristics of primary vascular smooth muscle cells (pVSMCs) isolated from rat aorta. (**a**) The growth of pVSMCs from the edge of the aortic rings (**b, c, d**) Characteristics of pVSMCs were confirmed using immunofluorescence staining and immunoblotting. More than 90% of cells were positive with the alpha-SMA, and it was well-maintained from passage 3 to passage 8

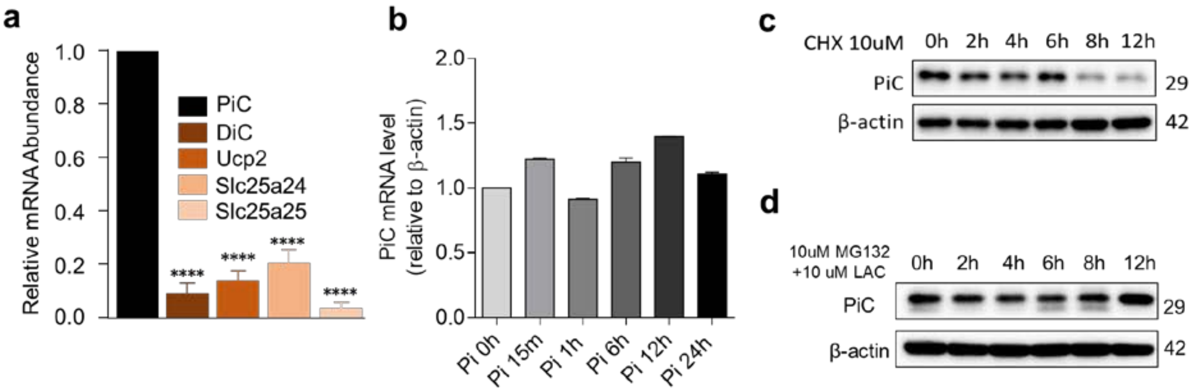

Supplementary Fig. 2. Transcriptional and translational regulation of mitochondrial Pi carrier (PiC). (**a**) Relative mRNA abundance of PiC, DIC, Ucp2, slc25a24, and slc25a25, indicating the highest expression of PiC in primary vascular smooth muscle cells (pVSMCs). (**b**) mRNA level of PiC after high Pi treatment in a time-dependent manner using real-time PCR. (**c, d**) Changes in PiC abundance in the presence of cycloheximide (CHX) or MG132 with lactacystin (LAC). Data were analyzed using one-way ANOVA followed by Tukey's comparison test. **** $P < 0.0001$.

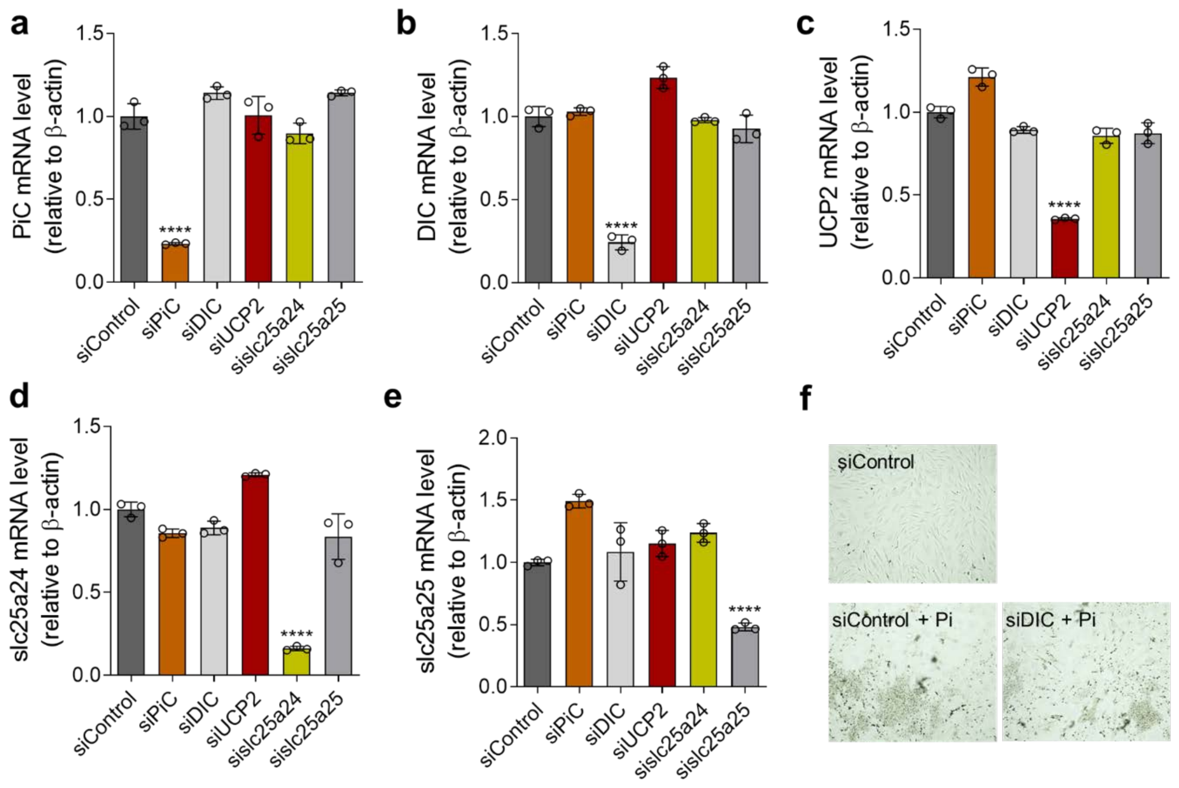

Supplementary Fig. 3. Silencing of mitochondrial Pi transporters in vascular smooth muscle cells. (**a–e**) Knockdown efficiencies were determined using real-time PCR showing the significant reduction of mRNA levels of target genes. (**f**) Effects of DIC knockdown on vascular calcification, which was estimated using von Kossa staining. Data were analyzed using one-way ANOVA followed by Tukey's comparison test. **** $P < 0.0001$.

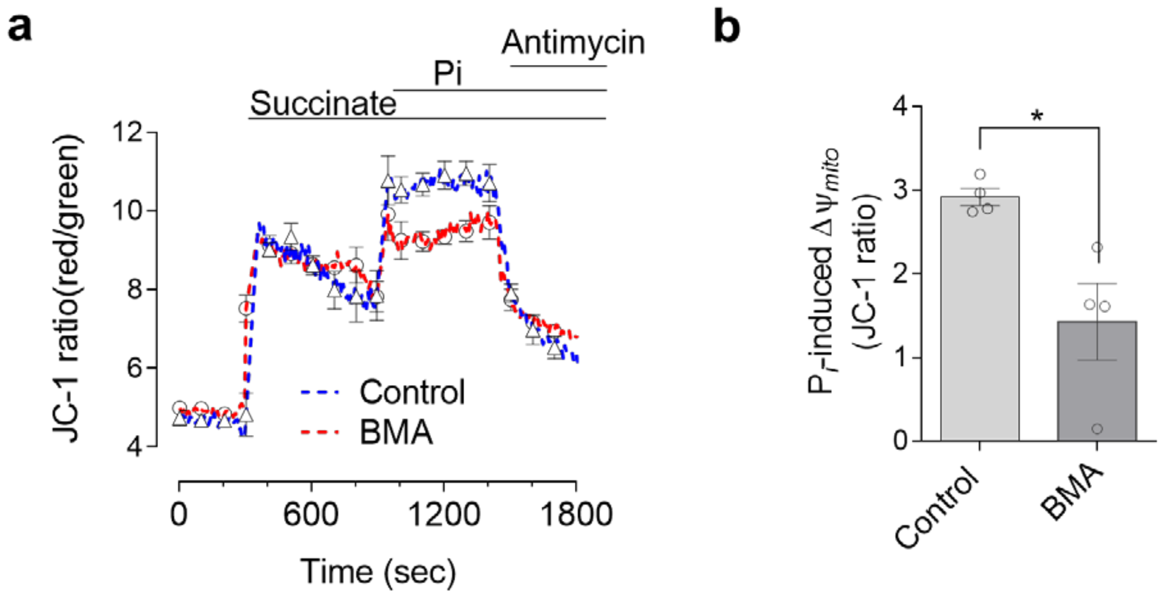

Supplementary Fig. 4. BMA blocks Pi-induced mitochondrial hyperpolarization. (**a, b**) The mitochondrial membrane potential of primary vascular smooth muscle cells with and without BMA pre-treatment was measured using JC-1 dye (# experiments; n=4). Data were analyzed using two-tailed Student's t-test. * $P < 0.05$.

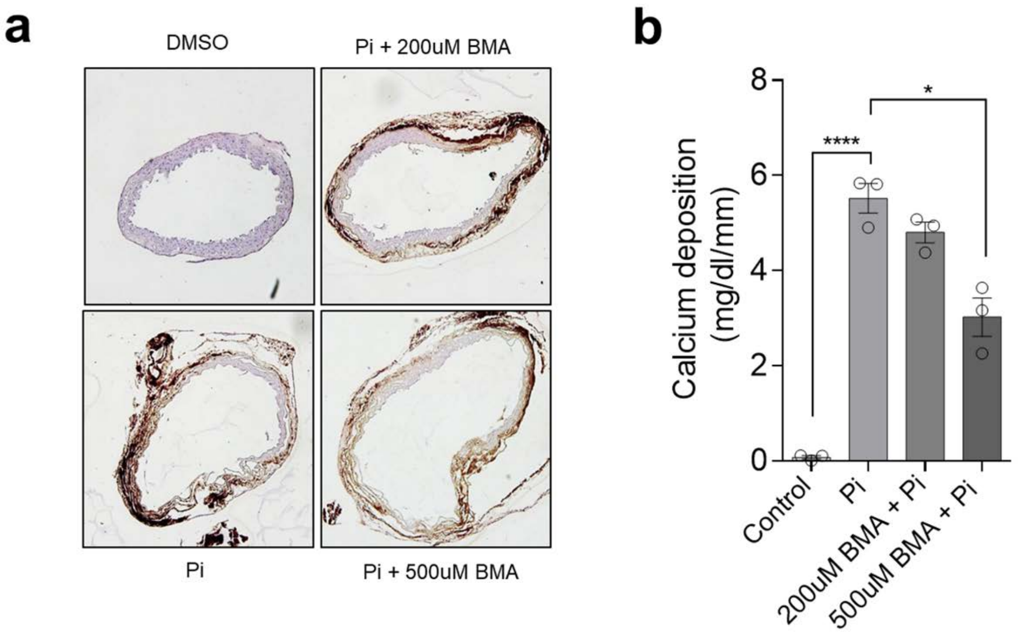

Supplementary Fig. 5. BMA inhibits Pi-induced calcification in rat aorta. (**a, b**) Rat aortic rings culture in the high Pi containing medium for seven days and the calcified areas were determined using von Kossa staining shown as the dark brown color (# experiments; n=3). Data were analyzed using one-way ANOVA followed by Tukey's comparison test. * $P < 0.05$; **** $P < 0.0001$.

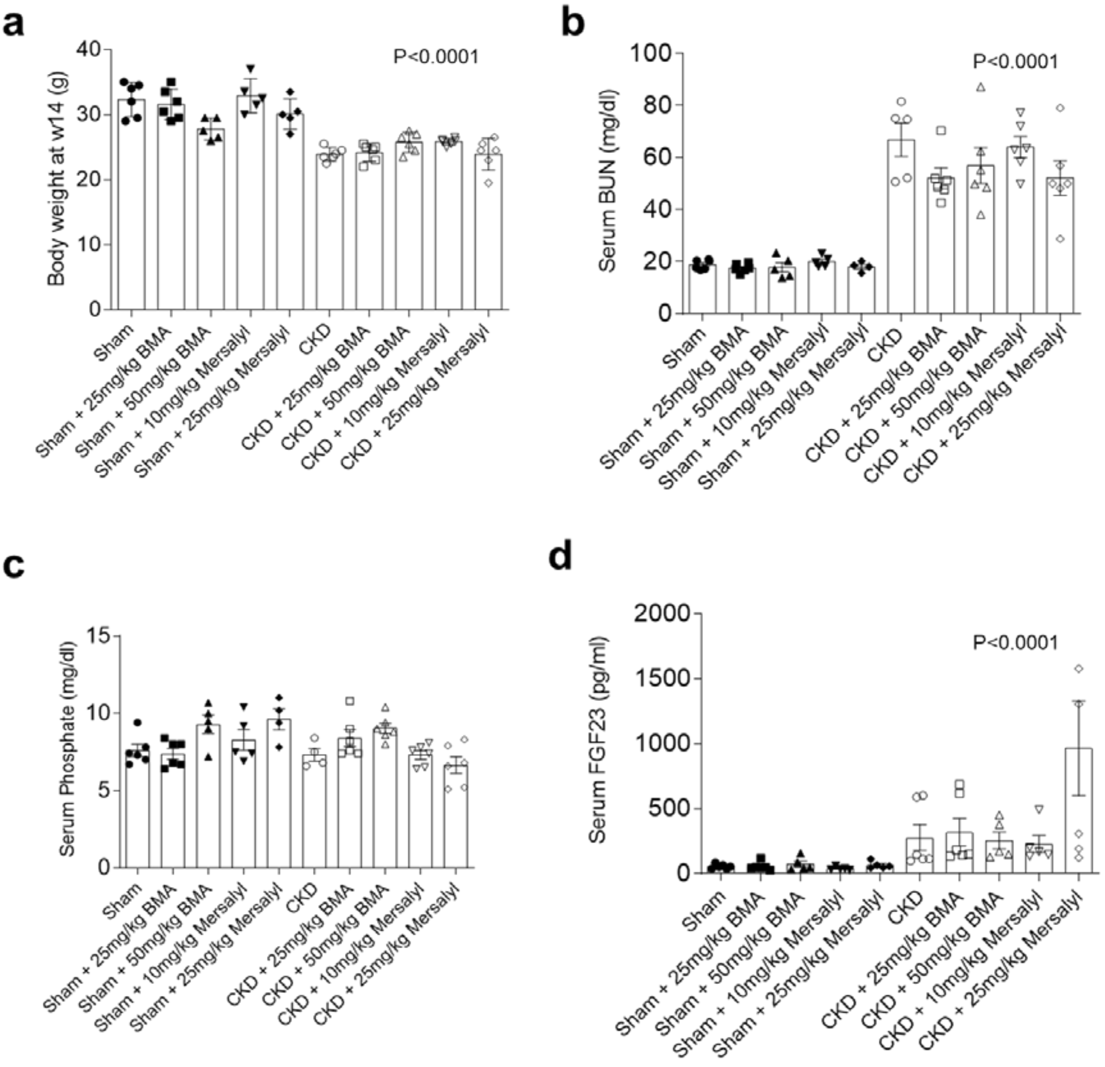

Supplementary Fig. 6. Body weight, blood urea nitrogen (BUN), serum phosphate, and FGF23 levels in CKD mouse models. (**a**) Body weight was measured every week to compare the difference among various groups. (**b–d**) Serum BUN, phosphate (Pi), and FGF23 were measured to estimate renal damage in mice models (# mice; n=5–6). Data were analyzed using one-way ANOVA followed by Tukey's comparison test.

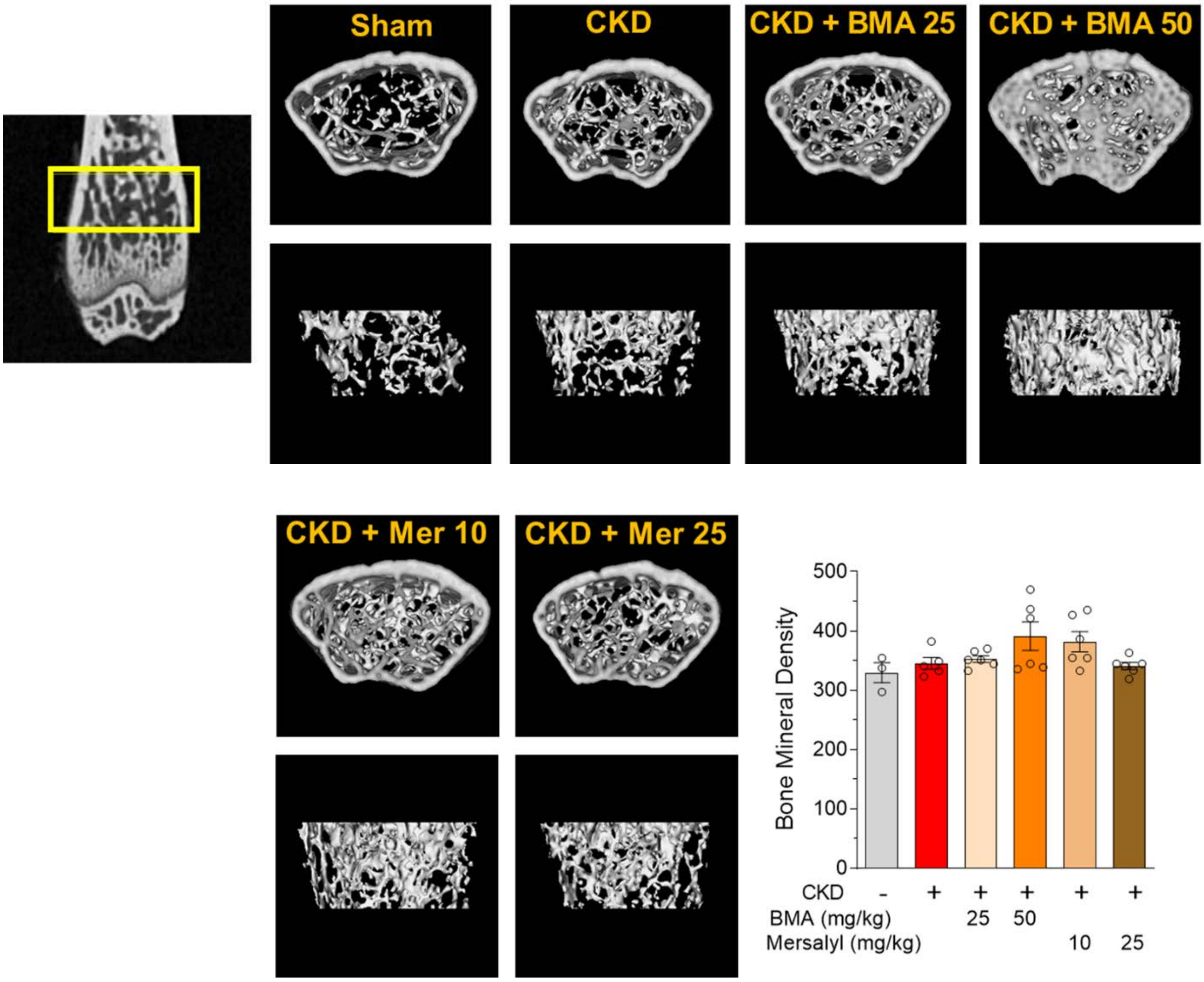

Supplementary Fig. 7. BMA or mersalyl does not affect the bone density of CKD mouse models. The micro-CT images of the femur head of CKD mouse treated with BMA or mersalyl were analyzed to calculate bone mineral density with the software.

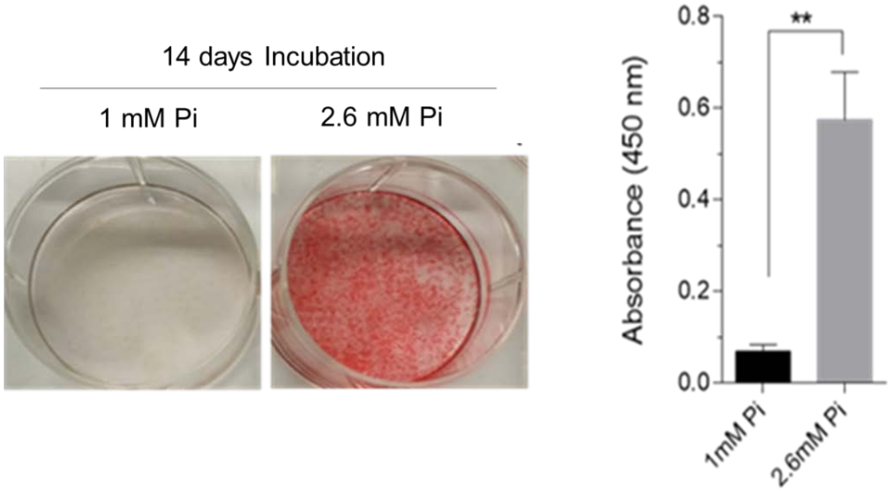

Supplementary Fig. 8. Long-term incubation induces calcific changes at lower Pi concentration. Rat primary vascular smooth muscle cells (pVSMCs) were incubated for 14 days with 2.6 mM Pi containing culture medium. Alizarin staining was used to estimate calcific changes in pVSMCs. Data were analyzed using the Student's t test ** $P < 0.01$.

Supplementary Table 1. Primer sequences for PCR

Target		5'-3' Sequences	Size (bp)
β-actin	**F**	ATGGTGGGTATGGGTCAGAA	119
	**R**	TCCATATCGTCCCAGTTGGT	
Runx2	**F**	GTTTGTTCTCTGACCGCCTC	150
	**R**	GCCTGGGATCTGTAATCTGA	
OPN	**F**	CTCGGATGAATCTGACGAAT	140
	**R**	GTCCGTAAGCCAAGCTATCA	
ALP	**F**	TGGACCTCATCAGCATTTGG	150
	**R**	TCAACTCATACTGCATGTCC	
Msx2	**F**	CTCTCGTCAAGCCCTTCGAG	113
	**R**	GGGGCTCATGTGTCTGG	
PiC	**F**	AGAGCAGCTGGTTGTGACAT	153
	**R**	ACACCTCTAAAGCCAAGCCT	
UCP2	**F**	ATGTGGTAAAGGTCCGCTTC	95
	**R**	CTCTCGTGCAATGGTCTTGT	
DIC	**F**	TCACCCACTTCCTCTCCAGT	106
	**R**	TGATACTCGCCCTTGGAGTT	
slc25a24	**F**	TAATGACGGGAAAATCGAGCCT	136
	**R**	TTCATTCCAGTCCACGGTCA	
slc25a25	**F**	AGGGGCAATGGCATCAATGT	82
	**R**	CAAGGCGTTTCATCTGCTCA	

F: Forward, **R**: Reverse, **bp**: base pairs

Western blots with membranes and markers

Figure 1c

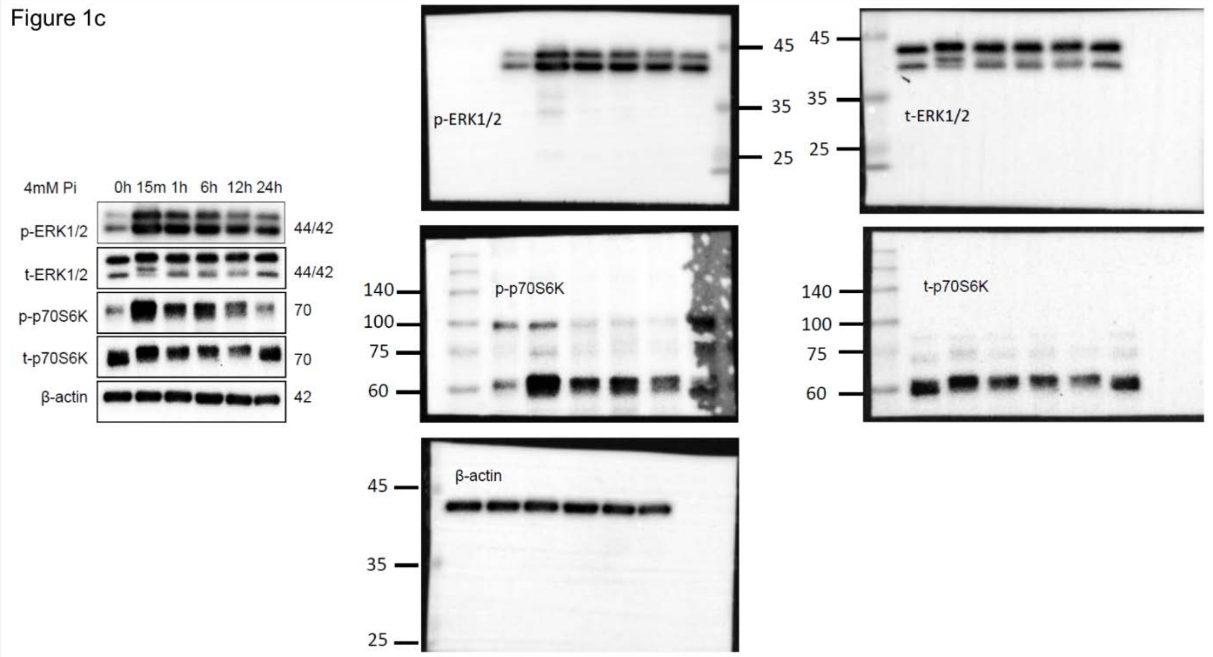

Figure 2b

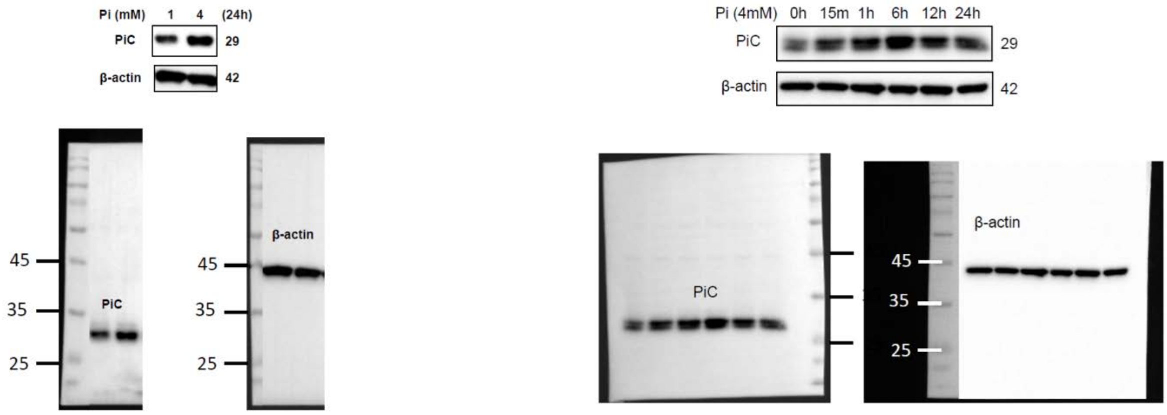

Figure 2c

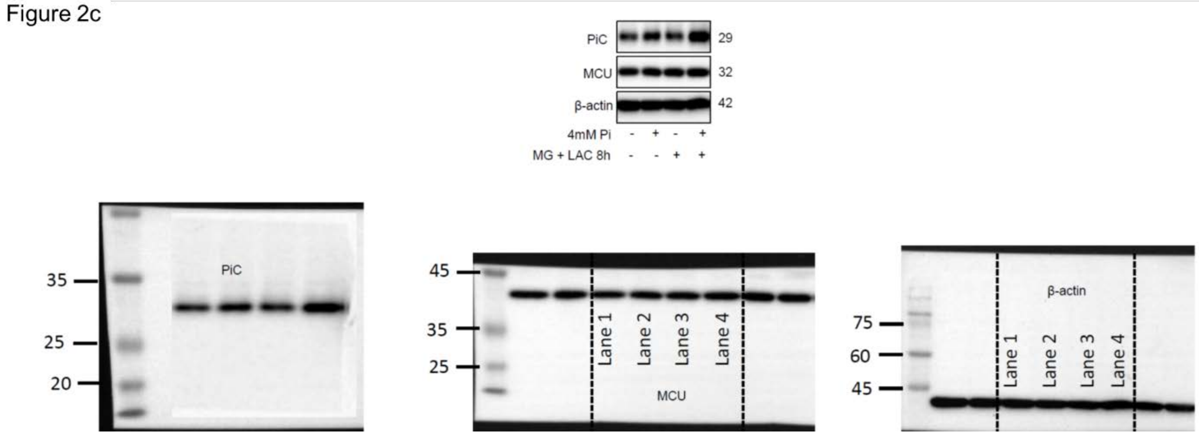

Figure 2d

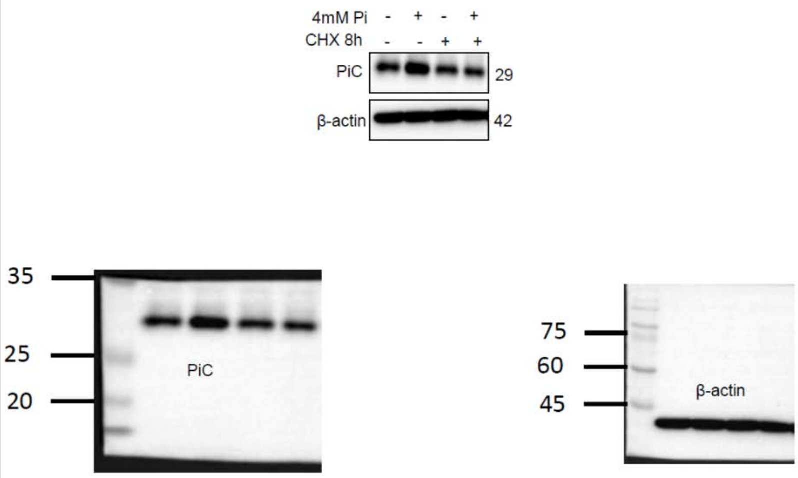

Figure S2

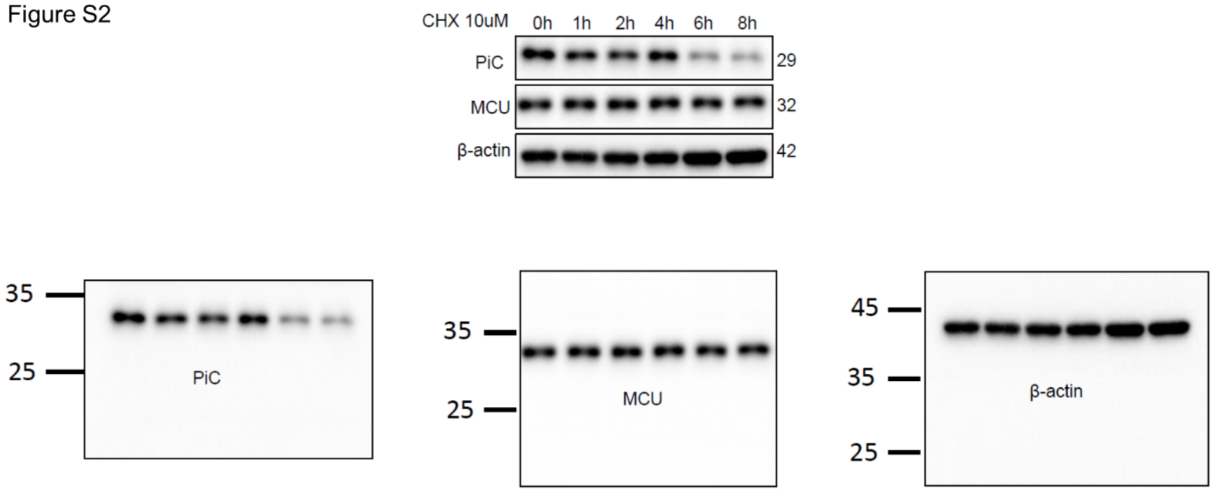

Figure 2e

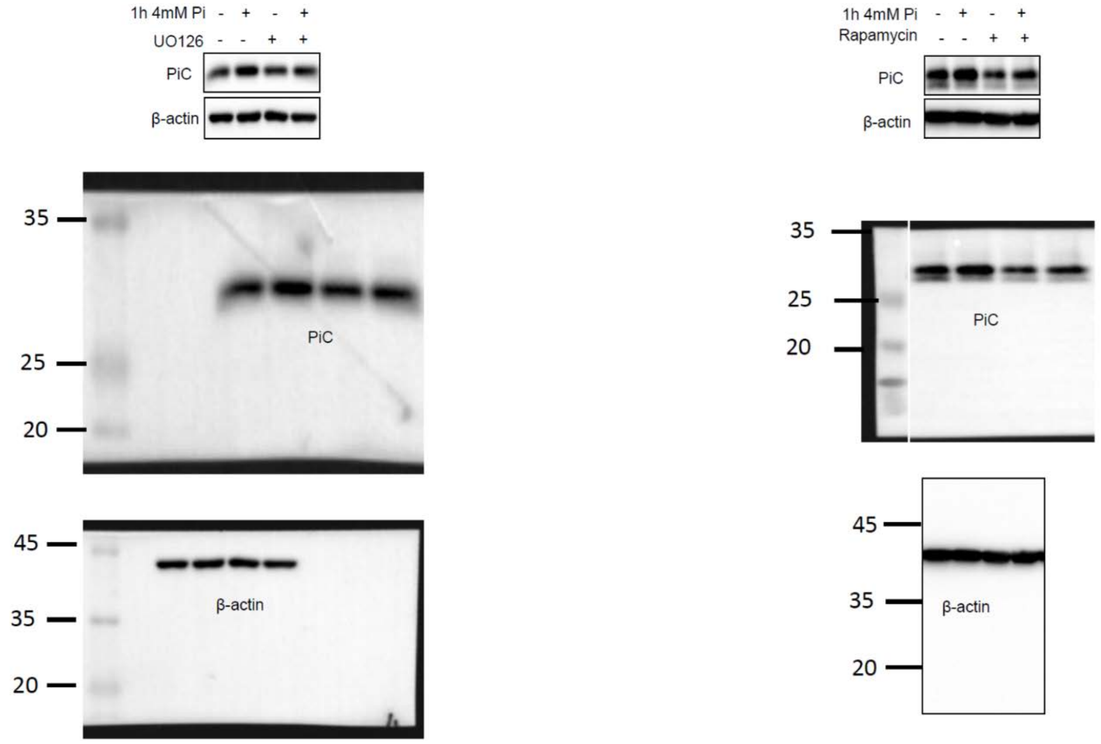

Figure 3c

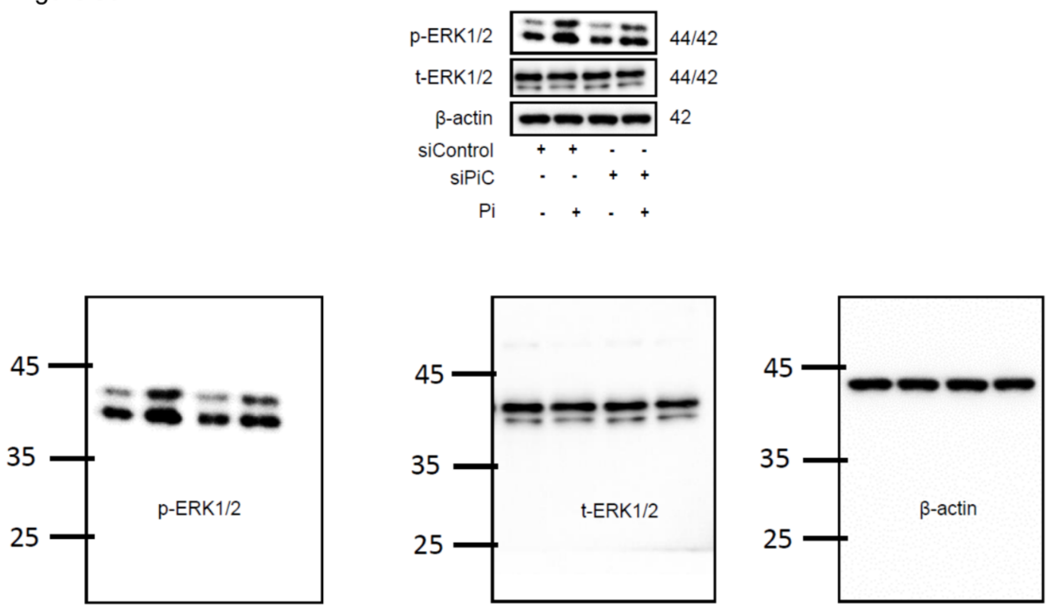

Figure 5c

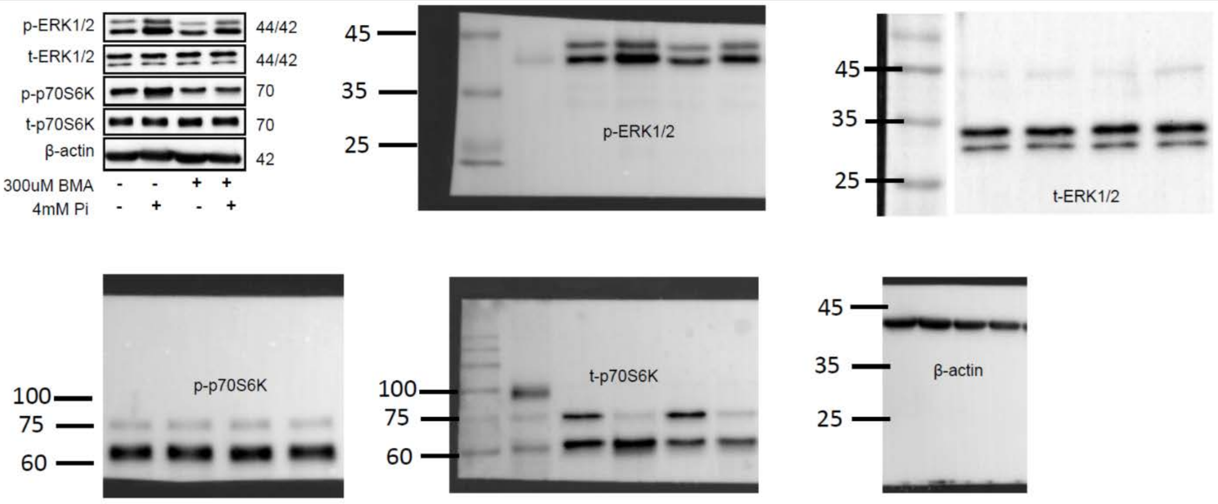

Supplement: Supplementary file 1 — Supplemental Information [file 12276_2023_950_MOESM1_ESM.pdf]
